# How Pre‐Pregnancy Weight and Polycystic Ovary Syndrome Impact Pregnancy Outcomes: A Population‐Based Cohort Study

**DOI:** 10.1002/hsr2.72088

**Published:** 2026-04-26

**Authors:** Elahe Sheklabadi, Marzieh Saei Ghare Naz, Maryam Mousavi, Maryam Rahmati, Seyed Alireza Ebadi, Fereidoun Azizi, Fahimeh Ramezani Tehrani

**Affiliations:** ^1^ Reproductive Endocrinology Research Center, Research Institute for Endocrine Molecular Biology, Research Institute for Endocrine Sciences Shahid Beheshti University of Medical Sciences Tehran Iran; ^2^ Department of Internal Medicine School of Medicine Shahid Beheshti University of Medical Sciences Tehran Iran; ^3^ Endocrine Research Center, Research Institute for Endocrine Disorders, Research Institute for Endocrine Sciences Shahid Beheshti University of Medical Sciences Tehran Iran; ^4^ Foundation for Research and Education Excellence Vestavia Hills Alabama USA

**Keywords:** adverse pregnancy outcomes, overweight/obesity, polycystic ovary syndrome

## Abstract

**Background and Aims:**

Pre‐pregnancy overweight/obese status among women with polycystic ovary syndrome (PCOS) could increase the risk of at least one adverse pregnancy outcome (APO). This study aimed to determine how pre‐pregnancy weight and PCOS impact pregnancy outcomes.

**Methods:**

For the current study, we used data collected in the Tehran Lipid and Glucose Study (TLGS), a cohort study with an average of 23 years of follow‐up, including 1105 women. The incidences of APOs, including gestational diabetes (GDM), preeclampsia (PE), and preterm birth, were compared between groups with different categories of pre‐pregnancy body mass index (18–24.9, 25–29.9, ≥ 30 kg/m^2^), PCOS, and non‐PCOS groups. The risk ratio (RR) and 95% confidence interval (95% CI) were estimated using the generalized linear models after adjusting for confounding factors. The participants included 864 non‐PCOS women and 241 women with PCOS.

**Results:**

Findings showed that the incidence rate of at least one APO among women with PCOS compared with non‐PCOS group was not significant (32.4% vs. 26.4%, *p* > 0.05). Obese women with PCOS (RR: 2.01; 95% CI: 1.11–3.62) and non‐PCOS (1.75; 1.10–2.79) were at increased risk of GDM compared to non‐PCOS normal weight women. Also, obese women with PCOS were at increased risk of PE (1.80; 1.08–2.97) compared to the non‐PCOS normal weight women. Overweight and obese women with PCOS were at increased risk of at least one APO (1.57; 1.06–2.32) and (1.44; 1.03–2.02), respectively.

**Conclusion:**

The study found that women with PCOS with pre‐pregnancy overweight/obese status are at increased risk of at least one APO and GDM. Management of pre‐pregnancy weight should be promoted in women with PCOS.

**Abbreviations**
17‐OHP: 17‐hydroxyprogesterone
AD: androstenedione
APOs: adverse pregnancy outcomes
BMI: body mass index
CKD: chronic kidney disease
DHEAS: dehydroepiandrosterone sulfate
DM: diabetes mellitus
ELISA: enzyme‐linked immunosorbent assay
FAI: free androgen index
GDM: gestational diabetes
HDL: high‐density lipoprotein
HTN: hypertension
LDL: low‐density lipoprotein
PCOS: polycystic ovary syndrome
PE: preeclampsia
ppBMI: pre‐pregnancy body mass index
PTB: preterm birth
SHBG: sex hormone binding globulin
TC: total cholesterol
TLGS: Tehran Lipid and Glucose Study
TT: total testosterone
WHO: World Health Organization

## 1 Introduction

Polycystic ovary syndrome (PCOS) is a common endocrine and metabolic disorder in women of reproductive age [1, 2]. The clinical/biochemical hyperandrogenism, oligo‐ovulation or anovulation, and polycystic ovarian morphology on ultrasound are considered the main features of PCOS [3]. Evidence suggests that PCOS is more prevalent in overweight/obese women [4]. This syndrome is often considered a weight‐related disease that can lead to health problems such as metabolic syndrome, type 2 diabetes, cardiovascular disease, mental disorders, endometrial cancer, and infertility [5, 6, 7, 8, 9]. According to the World Health Organization (WHO) report in 2016, more than 1.9 billion adults worldwide were overweight, and of this number, more than 650 million were obese [10]. Along with the rising trend of obesity, PCOS is also on the rise [11].

Recent meta‐analyses showed that women with PCOS are at increased risk of pregnancy‐induced hypertension (HTN), preeclampsia (PE), gestational diabetes (GDM), and preterm birth (PTB) [12, 13, 14, 15, 16]. Increasing maternal body mass index (BMI) before pregnancy is also associated with adverse pregnancy outcomes (APOs) as well as short‐term and long‐term complications for both the mother and the child [17, 18, 19]. For every 5–7 kg/m² increase in pre‐pregnancy body mass index (ppBMI), the risk of PE might double [20]. The effects of ppBMI on perinatal outcomes in women with PCOS are not yet fully understood [21]. A limited number of studies conducted in China, Taiwan, South Korea, and Belgium have compared APOs in women with and without PCOS according to BMI [22, 23, 24, 25, 26]. While some studies suggest that obesity exacerbates the risk of certain complications like GDM, HTN during pregnancy, PE, and cesarean section in women with PCOS, other evidence paints a more nuanced picture [27, 28].

Moreover, the current body of research on this topic exhibits several notable limitations. Many of these studies have been constrained by a lack of population‐based sampling, instead relying on retrospective analyses of medical record data [23, 24, 25, 26]. Additionally, short follow‐up periods have prevented comprehensive longitudinal assessment of outcomes [23, 25]. Finally, the populations examined have often been limited to infertile women, potentially limiting the generalizability of findings [23, 24]. A meta‐analysis study considered APOs in PCOS as independent of obesity during pregnancy [29].

The present prospective cohort study sought to compare APOs between women with and without PCOS with a particular focus on the influence of ppBMI.

## 2 Methods

### 2.1 Study Design and Study Population

This population‐based study was conducted as a prospective cohort study in the context of the Tehran Lipid and Glucose Study (TLGS). The TLGS study began in 1998 and was conducted in district 13 of Tehran to investigate the prevalence, risk factors, and consequences of non‐communicable diseases, as well as the effects of therapeutic interventions on these diseases in a population over 3 years. Individuals in the study are followed up every 3 years and undergo examination and testing. Currently, information from 7 phases of the study is available (phase 1 from 1998 to 2001, phase 2 from 2001 to 2005, phase 3 from 2005 to 2008, phase 4 from 2008 to 2011, phase 5 from 2011 to 2014, phase 6 from 2014 to 2017, and phase 7 from 2017 to 2021). The previous study has reported the details of the methodology of TLGS [30, 31].

For the present study, we utilized comprehensive data collected on the obstetric and reproductive status of participants from initiation of the study (1998) and subsequent six follow‐up visits; details have been previously published [32]. Comprehensive data on demographic variables, obstetric history, and reproductive factors were collected via a standardized questionnaire administered during in‐person interviews. Additionally, general anthropometric measurements and physical examinations were conducted, including an assessment of hirsutism using the modified Ferriman‐Gallwey scoring system. A licensed general practitioner performed all physical examinations.

We defined PCOS in our study using the Rotterdam criteria. For the purposes of the current study, the study population was drawn from reproductive‐aged women between 18 and 45 years old who presented for baseline examination. From this cohort, we included only those participants whose PCOS status was definitively documented. This allowed for precise sub‐categorization of the cohort into PCOS and non‐PCOS groups. Additionally, eligible participants were required to have experienced at least one childbirth during the study period, with clearly documented pregnancy outcomes. Participants were excluded from the final study if they met any of the following criteria: history of multiple gestations, uncertainty regarding APOs in terms of GDM, PE, and PTB, or pre‐existing diabetes mellitus (DM) or HTN, chronic kidney disease (CKD) prior to the index pregnancy, parous at the commencement of the study, no experience of pregnancy during the study follow‐up period, ppBMI < 18 or > 40 kg/m^2^.

Considering GDM as the outcome and PCOS as the exposure variable, the total required sample size, calculated using the formula $N=\frac{{\left(z_{1-\alpha }+z_{1-\beta }\right)}^{2}}{{\rho \sigma }^{2}{\left(\ln\mathrm{RR}\right)}^{2}}\;$with a desired power of 80% (*Z*_1 − *β*_ = 0.84), a Type I error level of 0.05 (*Z*_1 − *α*_ = 1.64), a prevalence of GDM (*ρ*) of 12%, and a hazard ratio of 1.6 (the risk of developing GDM in obese women with a history of PCOS compared to the normal‐weight control group), and a variance (*σ* ^2^) of 0.25, was determined to be approximately 934 women.

**Figure 1:**
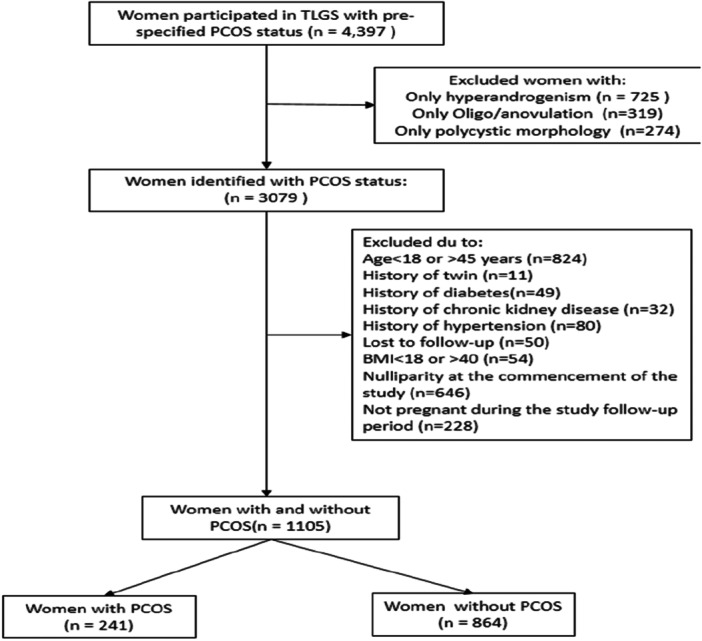
Study population flowchart. AOV, anovulation; BMI, body mass index; CKD, chronic kidney disease; DM, diabetes mellitus; HA, hyperandrogenism; HTN, hypertension; PCOM, polycystic morphology; PCOS, polycystic ovary syndrome; TLGS, Tehran Lipid and Glucose Study.

After applying the aforementioned inclusion and exclusion criteria, a total of 1105 women were ultimately included in the final analysis cohort. This comprised 241 participants with confirmed PCOS and 864 women without PCOS. Both groups were further stratified into three subgroups based on ppBMI: < 25, 25–29.9, and ≥ 30 kg/m². Figure 1 illustrates the study flowchart.

### 2.2 Definition of Variables

The primary objective of this study was to assess the impact of pre‐pregnancy weight and PCOS on pregnancy outcomes. The secondary objective was to compare the effect of pre‐pregnancy obesity on pregnancy outcomes between women with PCOS and those without PCOS.

According to the WHO definition, individuals with a BMI of 25–29.9 kg/m² are classified as overweight, while those with a BMI of ≥ 30 kg/m² are considered obese [10].

PCOS was defined by the presence of two out of three criteria (according to Rotterdam 2003 criteria), including clinical/biochemical hyperandrogenism, oligo‐ovulation or anovulation, and polycystic morphology of the ovaries on ultrasound, in the absence of other endocrine disorders [3]. Oligo/anovulation was defined as either regular or irregular menstrual cycles ≥ 34 days or a history of eight or fewer menstrual cycles in a year. The clinical symptoms of hyperandrogenism included hirsutism, which was diagnosed based on a standardized scoring system of the modified Ferriman‐Gallwey scale (≥ 8), acne, or androgenic alopecia. Biochemical hyperandrogenism was assessed as an increased level of one or more serum androgens, including dehydroepiandrosterone sulfate (DHEAS), testosterone, or androstenedione (AD), above the 95th percentile, determined in the selected healthy, non‐hirsute, eumenorrheic women in the study population.

APOs were self‐reported by participants using a standardized questionnaire. These self‐reported data were corroborated by participants' medical records. These outcomes were defined according to the national clinical guidelines utilized throughout the country's prenatal care services as follows: GDM was defined by fasting glucose levels ≥ 7.0 mmol/L and/or 2‐h glucose levels ≥ 7.8 mmol/L utilizing a 75 g oral glucose tolerance test in accordance with the specific criteria outlined in the 1999 WHO guideline [33, 34]. PE was characterized by identifying systolic blood pressure ≥ 140 mmHg and/or diastolic blood pressure ≥ 90 mmHg on two separate occasions, with a minimum interval of 6 h, occurring after the 20th week of gestational age [35, 36]. PTB was defined as a delivery before 37 weeks of gestational age, according to the WHO [37].

Physical activity was determined using the Modifiable Activity Questionnaire, which has been validated and has good reliability in the Iranian population [38]. This questionnaire measures physical activity related to leisure time, household activities, and occupational activities [39]. Adequate physical activity was defined as MET ≥ 600 min per week [40].

### 2.3 Biochemical, Hormonal, and Ultrasonographic Assessment

All blood samples were taken between 7:00 and 9:00 a.m. after 12 h of overnight fasting during the early follicular phase of spontaneous or progesterone‐induced menstrual cycle. All sera were stored at −80°C until the time of measurements. The total testosterone (TT), DHEAS, AD, and 17‐hydroxyprogesterone (17‐OHP) levels were measured using enzyme immunoassay by Diagnostic Biochem Canada Co., Ontario, Canada. The sex hormone‐binding globulin (SHBG) levels were measured using an enzyme‐linked immunosorbent assay (ELISA) by Canada Diagnostic Biochem Co., Ontario, Canada. All ELISA tests were performed using the Sunrise ELISA reader (Tecan Co., Salzburg, Austria). The intra‐ and inter‐assay coefficients of variation were 3.6% and 6% for TT, 1.9% and 3.2% for DHEAS, 1.1% and 4.1% for SHBG, and 1.9% and 3.4% for TSH, respectively. The free androgen index (FAI) was calculated by TT (nmol/L) × 100/SHBG (nmol/L).

Fasting blood sugar (FBS) was assessed using glucose oxidase by the enzymatic colorimetric method (Pars Azmoon kit, Iran, inter and intra assay < 2.2%). Total cholesterol (TC) was measured (enzymatic colorimetric method with cholesterol esterase and cholesterol oxidase). High‐density lipoprotein (HDL) was assayed after precipitation of the apolipoprotein B (apo B)‐containing lipoproteins with phosphotungstic acid. Triglycerides (TG) were assayed using glycerol phosphate oxidase. Intra‐ and inter‐assay coefficients of variations for TC, HDL‐C, and TG were below 1.9, 3, and 2.1%, respectively. Analyses were performed using related kits (Pars Azmon Inc., Tehran, Iran) and a Selecta 2 autoanalyzer (Vital Scientific, Spankeren, Netherlands). To calculate low‐density lipoprotein (LDL), the modified Friedewald equation was used [30]. The intra‐ and interassay CVs were both 2.2% for glucose. Intra and interassay CVs were 0.6% and 1.6% for TG, respectively. For both total and HDL, intra‐ and interassay CVs were 0.5% and 2%, respectively. All laboratory tests adhered to established protocols, which are elaborated elsewhere in detail [30, 31].

Transvaginal or transabdominal ovarian ultrasound examinations of the study participants were conducted using a 3.5‐MHz transabdominal transducer and a 5‐MHz transvaginal transducer, both operated by an experienced sonographer. The virgin patients underwent transabdominal ultrasound. The ultrasound examinations were performed on the same day as the blood samples were collected. The SONOACE R3 is a high‐quality portable ultrasound system designed (Product name: USS‐SAR3N2R/WR) was used to provide high‐performance medical imaging.

A composite variable means someone has experienced at least one of the APOs, regardless of the type of APO.

### 2.4 Ethical Considerations

All adult participants provided their informed consent by signing forms that followed the basic principles of the Declaration of Helsinki. The study was approved by the ethical review board of the Research Institute for Endocrine Sciences, Shahid Beheshti University of Medical Sciences, Tehran, Iran (Ethic code: IR.SBMU.ENDOCRINE.REC.1401.091).

### 2.5 Statistical Analysis

Descriptive statistical indices, including mean (standard deviation) and median (interquartile range), were presented to examine continuous variables, with and without normal distribution, respectively. The assumption of normality was tested using the Kolmogorov–Smirnov test, and parametric (*t*‐test) and non‐parametric (Mann–Whitney *U*‐test) tests were used to examine differences between the PCOS and non‐PCOS groups. Categorical variables were reported as numbers (percentages) and were compared between the two groups using the Chi‐square test or Fisher's exact test.

It should be noted that APO variables were considered as GDM, PE, PTB, and composite (at least one APO) as the main responses of this study. The generalized linear models were used to assess the association between PCOS and APOs; the risk ratios (RRs) were reported along with 95% confidence intervals (CIs) adjusted for age, physical activity, family history of diabetes, and family history of cardiovascular disease, smoking, and LDL density as confounding variables. The presence of multicollinearity among the confounding variables was assessed by calculating the variance inflation factor for each variable. Furthermore, an interaction term between PCOS and ppBMI categories (normal, overweight, obese) was included in the models to assess the association between PCOS and pregnancy outcomes according to ppBMI categories. Forest plots were used to visualize the adjusted RRs of APOs based on ppBMI regardless of PCOS status and based on the PCOS status regardless of ppBMI categories.

In this study, the non‐PCOS group with ppBMI < 25 kg/m^2^ was considered as the reference group.

The R software version 4.2.2 was used for analysis, and all statistically significant findings were derived from two‐tailed statistical tests and reported with *p* values less than 0.05.

To assess the power of our study based on the sample size and the effect sizes for the RRs related to the most significant interactions between PCOS and ppBMI categories, a post hoc power analysis was conducted with a significance level of 0.05. The analysis indicated a power of 0.9, suggesting that the study is adequately powered to detect significant effects of the interaction between PCOS status and BMI categories on APOs.

## 3 Result

In this study, 241 women with PCOS and 864 women without PCOS were included. The demographic, clinical, and laboratory characteristics of study participants according to their PCOS status are presented in Table 1. The median maternal age and ppBMI of women with PCOS were 27 (IQR: 22–33) years and 25.63 (IQR: 22.83–29.30) kg/m^2^, and in the non‐PCOS group were 30 (IQR: 24–37) years and 25.36 (22.36–28.47) kg/m^2^, respectively. There were no statistically significant differences in various demographic, anthropometric, and metabolic parameters of women with and without PCOS. There were significant differences between the two groups in FBS levels and levels of androgens (TT, DHEAS, SHBG, FAI) (Table 1).

**Table 1:** Demographic, clinical, and laboratory characteristics of the study based on the PCOS status of participants.

Variables	PCOS (*n* = 241)	Non PCOS (*n* = 864)	*p* value
Age (year)^a^	27 (22–33)	30 (24–37)	< 0.001
Age groups^b^			
18–29 years	148 (61.4)	413 (47.8)	< 0.001
30–45 years	93 (38.6)	451 (52.2)	
Years of formal education^b^			
< 12 years	211 (87.6)	754 (87.3)	
≥ 12 years	30 (12.4)	110 (12.7)	0.90
Adequate physical activity (Yes)^b^	82 (34.0)	277 (32.1)	0.56
Cigarette smoking (Yes)^b^	6 (2.5)	27(3.1)	0.60
SBP (mmHg)^a^	107 (100–114) 107	108 (101–114)	0.22
DBP (mmHg)^a^	71 (66–78)	72 (67–79)	0.33
BMI (kg/m²)^a^	25.63 (22.83–29.30)	25.36 (22.36–28.47)	0.23
BMI groups^b^			
18–24.9 (kg/m²)	113 (46.9)	418 (48.4)	0.20
25–29.9 (kg/m²)	78 (32.4)	308 (35.6)	
≥ 30 (kg/m²)	50 (20.7)	138 (16.0)	
Waist circumference (cm)^a^	83 (75–91)	81 (73–89)	0.25
Waist‐to‐hip ratio^a^	0.80 (0.75–0.85)	0.80 (0.75–0.85)	0.88
At least one birth^b^			
No	97 (40.2)	270 (31.2)	0.009
Yes	144 (59.8)	594 (68.8)	
FBS (mg/dL)^a^	84.00 (80.00–90.00)	86.00 (81.00–91.00)	0.02
Total cholesterol (mg/dL)^a^	177 (153.00–206.50)	183 (159.00–206.00)	0.06
LDL cholesterol (mg/dL)^a^	110.10 (89.00–131.40)	113.9 (93.65–135.35)	0.05
HDL cholesterol (mg/dL)^a^	42.00 (35.00–49.00)	44.00 (39.00–52.00)	0.10
Triglycerides (mg/dL)^a^	101.00 (71.50–143.50)	100.00 (74.00–145.00)	0.75
Total testosterone (ng/mL)^a^	0.58 (0.33–0.81)	0.28 (0.12–0.54)	< 0.001
DHEAS (µg/dL)^a^	150.79 (89.21–211.66)	119.68 (73.03–179.35)	< 0.001
Androstenedione (ng/mL)^a^	1.84 (1.09–2.30)	1.20 (0.90–1.70)	< 0.001
SHBG (nmol/L)^a^	48.00 (36.50–65.45)	53.00 (41.20–75.35)	0.002
Free androgen index^a^	1.11 (0.69–1.76)	0.49 (0.22–0.96)	< 0.001
17‐OHP (ng/mL)^a^	1.65 (1.10–2.14)	1.45 (1.00–2.10)	0.23
TSH (mIU/L)^a^	1.96 (1.19–3.37)	2.06 (1.49–3.38)	0.20

*Note:* Quantitative variables are reported as median (first quartile‐third quartile) and qualitative variables as number (percentage).

Abbreviations: 17‐OHP, 17‐hydroxyprogesterone; BMI, body mass index; DBP, diastolic blood pressure; DHEAS, dehydroepiandrosterone sulfate; FBS, fasting blood sugar; HDL, high‐density lipoprotein; LDL, low‐density lipoprotein; SBP, systolic blood pressure; SHBG, sex hormone binding globulin; TSH, thyroid‐stimulating hormone.

^a^ Mann–Whitney *U*‐test.

^b^ Chi‐square test.

The incidence rates of GDM, PE, PTB, and at least one APO among women with PCOS compared with the non‐PCOS group were (14.5% vs. 12.7%), (16.6% vs. 12.6%), (6.6% vs. 4.9%), and (32.4% vs. 26.4%), respectively, *p* > 0.05. According to obese status, obese women, compared to non‐obese women, regardless of PCOS, had a higher incidence rate of GDM (22% vs. 12.6%) and at least one APO (38% vs. 30.9%), *p* > 0.05. Also, across four groups defined by the presence or absence of PCOS and obesity, individuals with obese and non‐PCOS status had higher incidence rates of GDM, and individuals with PCOS and non‐obese status had higher incidence rates of PE. The incidence rates of PTB were approximately similar in four groups, while the incidence rate of the composite variable (at least one APO) was higher in the PCOS and obese groups (Figures S1 and S2).

**Figure 2:**
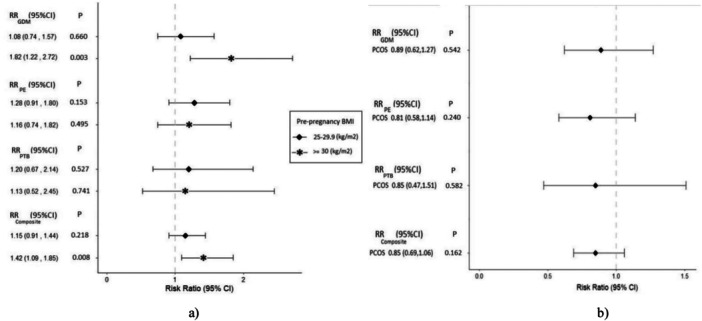
Forest plots of adjusted risk ratio (95% CI) of adverse pregnancy outcomes (a) based on pre‐pregnancy BMI regardless of PCOS status (b) based on the PCOS status regardless of pre‐pregnancy BMI status.

Regardless of PCOS status, the risk of GDM in obese women was increased by 82% (RR: 1.82; 95% CI: 1.22–2.72, *p* = 0.003) compared to the reference group, after adjustment for potential confounders, including age, physical activity, family history of diabetes, family history of cardiovascular disease, smoking, and LDL density (Figure 2a). Additionally, there was no significant increased risk of PE among obese women (RR: 1.16; 95% CI: 0.74–1.82, *p* = 0.495) and overweight women (RR: 1.28; 95% CI: 0.91–1.80, *p* = 0.153) compared to the reference group. Obese women (RR: 1.13; 95% CI: 0.52–2.45, *p* = 0.741) and overweight women (RR: 1.20; 95% CI: 0.67–2.14, *p* = 0.527) also did not have an increased risk of PTB. However, obese women were at increased risk of at least one APO (composite variable) compared to the reference group (RR: 1.42; 95% CI: 1.09–1.85, *p* = 0.008) (Figure 2a).

Figure 2b revealed that regardless of the impact of ppBMI, women with PCOS were not at increased risk of GDM (RR: 0.89; 95% CI: 0.62–1.27, *p* = 0.542), PE (RR: 0.81; 95% CI: 0.58–1.14, *p* = 0.240), and PTB (RR: 0.85; 95% CI: 0.47–1.51, *p* = 0.582) and at least one APO (RR: 0.85; 95% CI: 0.69–1.06, *p* = 0.162) compared to non‐PCOS women in adjusted models for mentioned variables.

Table 2 demonstrated that there was no significant increased risk of APOs in all groups in the unadjusted model. However, after accounting for the potential interaction between PCOS status and ppBMI categories in the fully adjusted models, obese women with PCOS, as well as non‐PCOS women with obesity, demonstrated a significantly increased risk of GDM compared to the reference group (RR: 2.01; 95% CI: 1.11–3.62, *p* = 0.020) and (RR: 1.75; 95% CI: 1.10–2.79, *p* = 0.018), respectively. And, for risk of PE, just obese women with PCOS were at increased risk (RR: 1.80; 95% CI: 1.08–2.97, *p* = 0.022) compared to the reference group. The fully adjusted interaction model, or PTB, showed no significant increased risk for any group. However, obese women with PCOS (RR: 1.57; 95% CI: 1.06–2.32, *p* = 0.022) and overweight women with PCOS (RR: 1.44; 95% CI: 1.03–2.05, *p* = 0.031) were at increased risk of at least one APO.

**Table 2:** Unadjusted and adjusted risk ratio (95% CI) of the interaction impact of PCOS status and BMI categories on adverse pregnancy outcomes.

Models	Interaction effect	GDM		PE		PTB		Composite	
		RR (95% CI)	*p* value		*p* value		*p* value		*p* value
Model 1	BMI_(18–24.9)_ × non‐PCOS	1 (ref)		1 (ref)		1 (ref)		1 (ref)	
	BMI_(25–29.9)_ × non‐PCOS	1.03 (0.48, 2.20)	0.930	1.28 (0.70, 2.36)	0.415	2.31 (0.78, 6.82)	0.127	1.30 (0.85, 1.99)	0.211
	BMI_(≥ 30)_ × non‐PCOS	1.77 (0.86, 3.63)	0.116	0.75 (0.31, 1.78)	0.519	1.35 (0.33, 5.45)	0.668	1.39 (0.87, 2.20)	0.169
	BMI_18–24.9)_ × PCOS	0.94 (0.54, 1.65)	0.845	0.78 (0.47, 1.27)	0.327	1.24 (0.48, 3.19)	0.651	0.95 (0.68, 1.34)	0.811
	BMI_(25–29.9)_ × PCOS	0.94 (0.52, 1.68)	0.844	0.79 (0.47, 1.33)	0.383	0.95 (0.34, 2.61)	0.927	0.89 (0.62, 1.28)	0.562
	BMI_(≥ 30)_ × PCOS	1.46 (0.79, 2.67)	0.218	0.81 (0.44, 1.49)	0.518	0.98 (0.30, 3.13)	0.976	1.10 (0.74, 1.64)	0.604
Model 2	BMI_(18–24.9)_ × non‐PCOS	1 (ref)		1 (ref)		1 (ref)		1 (ref)	
	BMI_(25–29.9)_ × non‐PCOS	1.08 (0.70, 1.64)	0.71	1.20 (0.80, 1.80)	0.36	0.88 (0.44, 1.76)	0.73	1.05 (0.81, 1.37)	0.667
	BMI_(≥ 30)_ × non‐PCOS	1.75 (1.10, 2.79)	0.018	1.27 (0.75, 2.12)	0.36	0.97 (0.39, 2.43)	0.96	1.34 (0.98, 1.83)	0.056
	BMI_18–24.9)_ × PCOS	1.01 (0.58, 1.76)	0.96	1.22 (0.74, 1.99)	0.42	0.74 (0.28, 1.91)	0.53	0.99 (0.70, 1.39)	0.975
	BMI_(25–29.9)_ × PCOS	1.10 (0.58, 2.09)	0.75	1.06 (0.48, 2.36)	0.87	1.91 (0.88, 4.15)	0.10	1.44 (1.03, 2.05)	0.031
	BMI_(≥ 30)_ × PCOS	2.01 (1.11, 3.62)	0.020	1.80 (1.08, 2.97)	0.022	1.22 (0.37, 3.97)	0.73	1.57 (1.06, 2.32)	0.022

*Note:* Model 1: Unadjusted model. Model 2: Adjusted for age, physical activity, family history of diabetes, family history of cardiovascular disease, smoking, and low‐density lipoprotein density.

Abbreviations: APOs, adverse pregnancy outcomes; BMI, body mass index; GDM, gestational diabetes; PE, preeclampsia; ppBMI, pre‐pregnancy body mass index; PTB, preterm birth; RR, risk ratio.

**Figure 3:**
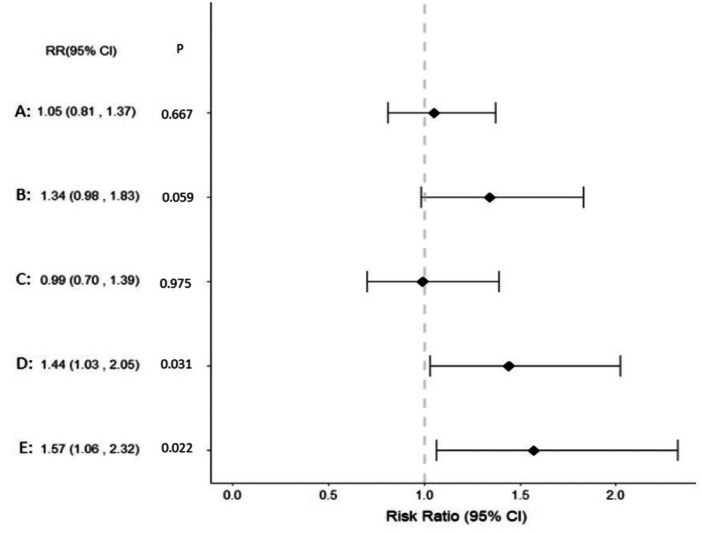
Forest plots of adjusted risk ratio (95% CI) of the interaction impact of PCOS status and BMI categories on the composite variable. A: Risk ratio of BMI 25–29.9 (kg/m^2^) × non‐PCOS; B: Risk ratio of BMI ≥ 30 (kg/m^2^) × Healthy; C: Risk ratio of BMI 18–24.9 (kg/m^2^) × PCOS; D: Risk ratio of BMI 25–29.9 (kg/m^2^) × PCOS; E: Risk ratio of BMI ≥ 30 (kg/m^2^) × PCOS. Reference category in all risk ratios: BMI 18–24.9 (kg/m^2^) × non‐PCOS.

The interaction analysis results revealed an interaction effect between BMI and PCOS on the development of at least one APO. Figure 3 shows forest plots of the adjusted RR (95% CI) of the interaction between PCOS status and BMI categories on the composite variable.

## 4 Discussion

This population‐based study aimed to determine whether pre‐pregnancy weight and PCOS impact pregnancy outcomes. Findings showed that the incidence rate of at least one APO among women with PCOS compared with the non‐PCOS group was 32.4% versus 26.4%. Regardless of PCOS status, the risk of APOs in obese women compared to women with normal weight has increased by 82%. However, among women with BMI ≥ 30 kg/m^2^, the risk of at least one APO increased by 42% compared to the normal ppBMI group. Women with PCOS and a ppBMI ≥ 30 kg/m^2^ almost two times higher than non‐PCOS group with a ppBMI < 25 kg/m^2^ were at risk of GDM and PE. Also, women with PCOS and ppBMI ≥ 30 kg/m^2^ and ppBMI 25–29.9 were 57% and 44% at increased risk of at least one APO. The interaction analysis results revealed an interaction effect between ppBMI and PCOS in the development of at least one APO.

The results of this study demonstrated that overweight and obese women, regardless of their PCOS status, were at an increased risk of developing GDM. In agreement with our findings, a systematic review and meta‐analysis have demonstrated that pre‐pregnancy overweight and obesity are associated with a 23% increased risk of GDM among women [41]. In contrast, a study conducted among Thai women indicated that pre‐pregnancy obesity did not significantly elevate the risk of APOs, including GDM and PE [42]. This discrepancy suggests that the relationship between pre‐pregnancy obesity and pregnancy complications may be influenced by additional factors, such as metabolic health status, genetic predispositions, racial and ethnic background of individuals.

We observed that PCOS, when analyzed without accounting for ppBMI, was not significantly associated with APOs. A two‐phase cohort study conducted by Wei Zheng and colleagues in China demonstrated that in subgroups of overweight and obese women, there was no significant difference in the prevalence of GDM between the PCOS and non‐PCOS groups (*p* > 0.05). That study is limited by using recorded medical data and not adjusted for potential confounding variables [25]. Another retrospective cohort study by Qiwei Liu and colleagues was conducted in China, involving 1357 women with PCOS and 6940 women without PCOS. The findings of that study indicated that PCOS was significantly associated with an increased incidence of gestational HTN, GDM, severe PE, and PTB (*p* ˂ 0.05). Obesity in women with PCOS was associated with a fourfold and 2.6‐fold increased risk of gestational HTN and GDM, respectively, compared to non‐obese women with PCOS. That study was limited by its use of recorded data and failure to adjust for potential confounding variables, such as ART and age [26]. In our study, the absence of a significant difference (*p* ˂ 0.05) in the incidence of APOs between individuals with PCOS and the non‐PCOS group, as well as the lack of an observable effect of ppBMI on the relationship between PCOS and APOs, may be attributable to specific characteristics of the study population. Additionally, the mild phenotype of PCOS observed in our cohort could have further influenced these findings. Mild phenotype, which is characterized by the absence of hyperandrogenism and is generally considered a less severe form of PCOS. Individuals with milder forms of the syndrome may exhibit fewer complications compared to those with more severe manifestations. Furthermore, the population‐based design of our study, as opposed to a patient‐based approach, may have introduced additional variability that affected the outcomes.

In the present study, we found that women with PCOS and pre‐pregnancy obesity were almost two times more likely than the non‐PCOS group with a ppBMI < 25 kg/m^2^ to be at risk of GDM. Also, women with PCOS and pre‐pregnancy overweight and obesity status were at increased risk of at least one APO. An association between PCOS and increased risk of PTB, particularly extremely PTB (< 28 weeks of gestation), was reported by several studies, which remained significant even after adjusting for potential confounders like obesity (*p* ˂ 0.05) [43, 44]. In a retrospective cohort study performed by V. De Frène and colleagues in Belgium from 2000 to 2009, 93 overweight women with PCOS (BMI ≥ 25 kg/m²) were compared to 107 normal‐weight women with PCOS (BMI < 25 kg/m²). The study observed that the incidence of PTB was significantly higher among overweight women compared to their normal‐weight counterparts (*p* ˂ 0.05). Data for that study were collected retrospectively from medical records obtained from various hospitals. However, the inclusion of women with infertility issues may have introduced a selection bias into the study design [24].

We found that women with PCOS and pre‐pregnancy obesity were almost two times more likely than the non‐PCOS group with a ppBMI < 25 kg/m^2^ to be at risk of PE. The association between hypertensive disorders of pregnancy and PCOS has been the subject of extensive research, revealing a significant link between the two conditions [14, 45, 46]. Women with PCOS are at an increased risk of developing hypertensive disorders during pregnancy, including gestational HTN and PE. Several potential mechanisms may explain that association, including insulin resistance, inflammatory processes, oxidative stress, hormonal imbalances, and the effects of obesity [47, 48].

The specific pathophysiological pathways through which PCOS exerts its deleterious effects on pregnancy outcomes remain a topic of debate. Proposed mechanisms include hyperandrogenism, hyperinsulinism, impaired decidualization, high luteinizing hormone levels, and low‐grade chronic inflammation, as well as various factors, including age, ART, ethnicity, and obesity [49, 50, 51, 52, 53, 54]. Furthermore, PCOS is regarded as an inflammatory disorder, exhibiting a low‐grade inflammatory state that is independent of obesity [55]. However, obesity can exacerbate the inflammatory status associated with pregnancy [56]. Additionally, the role of specific PCOS phenotypes and their differential impact on pregnancy risk has been the subject of investigation, with some studies suggesting that certain PCOS subtypes may confer a higher risk of pregnancy complications compared to others [57, 58, 59].

The strengths of this study include its population‐based design and prospective nature, with participants undergoing regular follow‐up assessments conducted at 3‐year intervals. The majority of participants conceived spontaneously without using ART. It is noteworthy that in women with PCOS, multiple pregnancies resulting from ART interventions may potentially exacerbate pregnancy complications and increase the risk of PTB. In the present study, cases involving multiple gestations were excluded from the statistical analysis. The limitations of this study include the absence of data pertaining to androgen levels during pregnancy, the lack of information on the exact gestational age at birth, and the absence of free testosterone measurement. Additional constraints involve relying on self‐reported pregnancy outcomes without active monitoring, insufficient sample size for certain outcomes due to their low prevalence, and inadequate sample size for comparing various phenotypes of PCOS and different weight categories. Our study is adequately powered with a sufficient sample size to reliably evaluate the risk of GDM and composite APOs in obese women with PCOS compared to normal‐weight women without PCOS. However, the sample size is insufficient to draw robust conclusions regarding other specific adverse outcomes, including PE, PTB, or to perform meaningful subgroup analyses, such as those stratified by PCOS and other ppBMI subgroups. Additionally, there's a lack of information on interventions and medications like metformin utilized by individuals with PCOS before and during pregnancy. Additionally, details concerning pregnancy weight gain were unavailable for analysis.

It is recommended that comprehensive cohort studies in populations with various ethnicities be conducted to compare APOs based on pre‐pregnancy obesity status and weight gain during pregnancy among individuals diagnosed with and without PCOS. Such studies should aim to elucidate the specific impact of both ppBMI and gestational weight gain on various pregnancy‐related complications, thereby enhancing our understanding of how weight management may influence maternal and fetal health outcomes. Furthermore, it is essential to evaluate both short‐term and long‐term pregnancy outcomes in offspring born to mothers with various PCOS phenotypes, particularly those who are obese.

## 5 Conclusion

The study found that women with PCOS with pre‐pregnancy overweight/obese status have an increased risk of at least one APO and GDM. Management of pre‐pregnancy weight should be promoted in women with PCOS.

## Author Contributions

All authors conceived of the study, participated in its design and helped to draft the manuscript. Likewise, all authors made suggestions and critical reviews to the initial draft and contributed to its improvement until reaching the final manuscript, which was read and approved by all authors.

## Ethics Statement

The study was approved by the ethical review board of the Research Institute for Endocrine Sciences, Shahid Beheshti University of Medical Sciences, Tehran, Iran, and informed consent was obtained from all participants. All methods were carried out in accordance with relevant guidelines and regulations, with the Declaration of Helsinki.

## Consent

The authors have nothing to report.

## Conflicts of Interest

The authors declare no conflicts of interest.

## Transparency Statement

The lead authors, Marzieh Saei Ghare Naz and Fahimeh Ramezani Tehrani, affirm that this manuscript is an honest, accurate, and transparent account of the study being reported; that no important aspects of the study have been omitted; and that any discrepancies from the study as planned (and, if relevant, registered) have been explained.

## Supporting information

Supporting File 1:

Supporting File 2:

## Data Availability

The data that support the findings of this study are available from the corresponding author upon reasonable request. The corresponding author had full access to all of the data in this study and takes complete responsibility for the integrity of the data and the accuracy of the data analysis.
