# Supplementary material for: How Pre‐Pregnancy Weight and Polycystic Ovary Syndrome Impact Pregnancy Outcomes: A Population‐Based Cohort Study
Source: Health Sci Rep. 2026 Apr 26;9(5):e72088. doi: 10.1002/hsr2.72088 (PMC13111921; doi:10.1002/hsr2.72088)
Supplement: Supplementary file 1 — Supporting File 1: [file HSR2-9-e72088-s001.docx]

The reproductive section of the TLGS questionnaire utilized for the purposes of the present study.

**TLGS phase:**

**Family ID:**

**Person ID:**

**Date of Interview** (DD/MM/YYYY):

Name: _______

Contact information: _______

Date of Birth (DD/MM/YYYY): _______

Marital Status: Single / Married /Widow/ Divorced

Education (years of study): _______

………………………………………………………………………………………………….

| 1. Have you ever received a diagnosis of any of the following conditions?(if yes please specify) | |
| --- | --- |
| 1- Type 2 Diabetes | 0-No□ 1-Yes□ |
| 2- Thyroid disorders | 0-No□ 1-Yes□ |
| 3- Hypertensive Disorders | 0-No□ 1-Yes□ |
| 4- Cardiovascular diseases, high blood pressure | 0-No□ 1-Yes□ |
| 5- Kidney diseases | 0-No□ 1-Yes□ |
| 6- Respiratory system diseases | 0-No□ 1-Yes□ |
| 7- Epilepsy | 0-No□ 1-Yes□ |
| 8- Hematologic diseases | 0-No□ 1-Yes□ |
| 9- Immune system diseases | 0-No□ 1-Yes□ |
| 10- Mental and psychological disorders | 0-No□ 1-Yes□ |
| 11- Digestive diseases | 0-No□ 1-Yes□ |
| 12- Chronic Infection such as tuberculosis, Malta fever | 0-No□ 1-Yes□ |
| 13- Polycystic ovary syndrome | 0-No□ 1-Yes□ |
| 14- Endometriosis | 0-No□ 1-Yes□ |
| 15- History of surgery due to gynecological problems  A-1- Uterine fibroid 2- Ovarian cyst 3- Endometriosis4- Fallopian tube adhesion5-Other | 0-No□ 1-Yes□ |
| 16- History of cancer | 0-No□ 1-Yes□ |

| 2. Have you regularly used any of the following medications for the treatment of your condition within the past six months? | |
| --- | --- |
| 1- Blood sugar lowering medications | 0- No□ 1- Yes, irregular□ 2- Yes, regular□ |
| 2- Thyroid disorder medications | 0- No□ 1- Yes, irregular□ 2- Yes, regular□ |
| 3- Blood pressure lowering medications or heart disease treatment | 0- No□ 1- Yes, irregular□ 2- Yes, regular□ |
| 4- Antidepressant-anxiety medications | 0- No□ 1- Yes, irregular□ 2- Yes, regular□ |
| 5- Corticosteroids | 0- No□ 1- Yes, irregular□ 2- Yes, regular□ |
| 6- Anticonvulsant medications | 0- No□ 1- Yes, irregular□ 2- Yes, regular□ |
| 7- Other (specify)…………………………………………. | 0- No□ 1- Yes, irregular□ 2- Yes, regular□ |

………………………………………………………………………………………………………………

3. Date of last menstrual cycle (DD/MM/YYYY):…………………..

4. Your age at the start of your first period: ........................ years

5. Have you experienced regular menstrual periods over the past year without the use of any medication?

1. Yes always □ 2-Yes most of the time □ 3-Sometimes □ 4-No rarely □ 5-No not at all □

6. If your menstrual cycles are regular due to medication, which of the following medications have you used most frequently?

6.1- Progesterone compounds:

0-No□ 1-Yes□

- 1. What was the total duration of use (in months) over the previous year?......

6.2- Combined oral contraceptive compounds:

0-No□ 1-Yes□

- 1. What was the total duration of use (in months) over the previous year?.............

6.3-Others, please specify …………………….

- 1. What was the total duration of use (in months) over the previous year?.................

7. Have your menstrual cycles been consistently regular since their onset?

1- Yes, always □

2- Yes □ my menstrual cycles were regular until the age of ….. years.

3- No, my menstrual cycles were irregular for approximately ……years before becoming regular.

4- No, my menstrual cycles have been irregular since their onset.□

8. On average, what is the duration (in days) of the interval between the onset of one menstrual period and the onset of the subsequent period?

1- With medication: ...................... days

2- Without medication:

1. Minimum................. days
2. Maximum ............... days
3. Average.............. days

9. Menstruation occurs only with medication: 0-No□ 1-Yes□

10. On average, how many days does your menstruation last? (Calculate the total of spotting and bleeding). ....................days

11. On average, how many sanitary pads do you use during menstruation? (In the entire period) ...................number

12. Continuous bleeding: 0-No□ 1-Yes□

13. On average, how many times do you menstruate per year?

1. Without taking medication: ...................... times

2- With taking medication: ...................... times

*Regularity of menstrual cycle according to the researcher: 0-Regular□ 1-Irregular□

……………………………………………………………………………………………..

14. Do you exhibit hirsutism or excessive coarse hair growth on your face, abdomen, arms, chest, or thighs?

0-No 1-Yes

15. If yes, have you used any specific drug treatment to prevent hair loss? Please specify…………………

16. What method do you use for hair removal? (Intervals to be updated)

1- Facial laser  (intervals) ---- Body  (intervals) -----

2- Facial electrolysis  (intervals) ---- Body  (intervals) ----

3- Facial waxing  (intervals) ---- Body  (intervals) -----

4-Facial razor  (intervals) ---- Body  (intervals) ----

5- Facial threading  (intervals) ---- Body  (intervals) -----

6-Facial tweezers  (intervals) ----- Body  (intervals) ----

7- Facial epilation  (intervals) ---- Body  (intervals) ----

8-Facial hair removal cream  (intervals) -----Body  (intervals) ----

9-Other: specify Face  (intervals) ----- Body  (intervals) -----

17. Has the severity of your hair growth changed over time?

1- It has improved ≤ 2- It has gotten worsened≤ 3- No, it has not changed 

0- Without medication ≤ 1- With medication or laser 

18. Have you experienced acne on your face, chest, or back?

0- No

1- Yes, only during puberty 

2- Yes, I continue to experience acne 

3- Yes, I didn't have acne previously, but have developed it recently 

4- Yes, but it has improved with treatment 

19. Assessment of hirsutism according to the Ferriman–Gallwey Score

(Rate hair growth in the following areas from 0 = none to 4 = extensive)

- Upper lip [0–4]………..
- Chin [0–4]…………..
- Chest [0–4]……….
- Upper abdomen [0–4]…………
- Lower abdomen [0–4]……….
- Upper arm [0–4]………..
- Thigh [0–4]………
- Upper back [0–4]………..
- Lower back [0–4]……..

Total score: ___ / 36

20. Rate acne severity according to the following categories:

**None:** No acne lesions present; **Mild:** Few comedones and small papules or pustules ; **Moderate:** Presence of larger inflammatory papules and pustules, possibly some cysts; **Severe:** Numerous inflammatory lesions, cysts, or nodules, widespread involvement

0. None 

1. Mild 

2. Moderate 

3. Severe

21. Rate the severity of scalp hair loss (alopecia) according to the following scale:

**Mild:** 20% or less scalp hair loss; **Moderate:** 21% to 49% scalp hair loss; **Severe:** 50% to 100% scalp hair loss

0. None 

1. Mild 

2. Moderate 

3. Severe

22. Have you experienced pregnancy at any time?

□ Yes

□ No

23.If yes, please continue:

Number of pregnancies: ___

Number of deliveries: ___

Number of live births: ___

Number of stillbirths: ___

Number of terminations: ___

24. Have you experienced any of the following adverse pregnancy outcomes during the study follow up? If yes, how many times?

- Preterm birth
  - □ Yes
  - □ No

…………

- Preeclampsia
  - □ Yes
  - □ No

………..

- Gestational diabetes

□ Yes

□ No

………….

- Intrauterine fetal demise (IUFD)
  - □ Yes
  - □ No

………..

- Placenta previa
  - □ Yes
  - □ No

………..

- Placental abruption
  - □ Yes
  - □ No

………..
