# Supplementary material for: How Pre‐Pregnancy Weight and Polycystic Ovary Syndrome Impact Pregnancy Outcomes: A Population‐Based Cohort Study
Source: Health Sci Rep. 2026 Apr 26;9(5):e72088. doi: 10.1002/hsr2.72088 (PMC13111921; doi:10.1002/hsr2.72088)
Supplement: Supplementary file 2 — Supporting File 2: [file HSR2-9-e72088-s002.docx]

(A) (B)

Supplementary Figure1. Incidence rates of adverse pregnancy outcomes based on (A) PCOS and non-PCOS status and (B) obese and non-obese.

Supplementary Figure 2. Incidence rates of adverse pregnancy outcomes based on PCOS and obesity status.
